# Comment on Jeon et al. Predictive Limitations of the Geriatric Trauma Outcome Score: A Retrospective Analysis of Mortality in Elderly Patients with Multiple Traumas and Severe Traumatic Brain Injury. *Diagnostics* 2025, *15*, 586

**DOI:** 10.3390/diagnostics15111350

**Published:** 2025-05-28

**Authors:** Miguel Velasco, Allen Chehimi, Jenny Chen, Marie Nour Karam, Afsheen Mansoori

**Affiliations:** 1 Department of Medical Education, South Bend Campus, Indiana University School of Medicine, South Bend, IN 46617, USA; migvela@iu.edu (M.V.); jc111@iu.edu (J.C.); markaram@iu.edu (M.N.K.); 2 Department of Medical Education, Detroit Campus, Michigan State University College of Human Medicine, Detroit, MI 48202, USA; achehim2@hfhs.org

**Keywords:** geriatric trauma, traumatic brain injury, geriatric trauma outcome score, predictive accuracy, multiple traumas, in-hospital mortality, older adults, transfusion, massive transfusion protocol, futility

The authors, Jeon et al., have provided an important observation that the Geriatric Trauma Outcome Score (GTOS) underestimates mortality at 24 h in elderly patients with polytrauma associated with severe traumatic brain injury (TBI) and concomitant extracranial hemorrhage [1]. This study positively contributes to the literature by reaffirming the need to capably define mortality at 24 h, especially in the context of the nation’s blood bank shortage following the COVID-19 pandemic [2,3]. Notably, a significant proportion of blood is often consumed in the first hours of trauma resuscitation for severely bleeding elderly patients with and without head injury [2,3,4]. The authors propose that more robust “futility criteria” are needed in order to define futility at earlier stages of resuscitation where the majority of blood products are used [1]. In their article, Jeon et al. anticipated the need for a tool that can reliably predict futility at the bedside within the first hours. Specifically, the authors have shown that the GTOS can be refined with the inclusion of the Glascow Coma Scale (GCS). By using GCS as a numeric value, the authors then modified the GCS and added it to the GTOS. This modified GTOS yielded greater accuracy as shown by its improved area under the curve performance [1].

In addition to the GTOS, which does not predict death until 24 h, other tools such as the Brain Injury Guidelines (BIGs) and frailty index, require the calculation of post-admission Injury Severity Score (ISS), which cannot be achieved at the bedside on admission. It has been proposed that by using this so-called frailty index, a traumatologist can prevent unnecessary interventions in patients while aggressively intervening in patients who would benefit from continued treatment. Therefore, the authors’ study confirms the need to find a triage tool for patients with TBI and associated severe hemorrhage which can predict early mortality [5,6].

Recently, because of the unintended consequence of the effect of balanced hemostatic resuscitation using 1:1:1 (red cells–plasma–platelets), with increasing quantities of whole blood, there have been attempts to define futility much earlier than the standard 24 h definitions. One such attempt is the STOP (Suspension of Transfusion and Other Procedures) criteria. These STOP criteria rely on markers which have 100% Positive Predictive Value (PPV) and specificity for predicting futility [7]. These markers, which are a combination of admission blood pressures, Return Of Spontaneous Circulation (ROSC), GCS before sedation and paralysis, lactic acid, and Thromboelastography (TEG) lysis, were developed from a mixed cohort of patients who not only had severe hemorrhage, but where some patients also had concomitant severe TBI [4]. As the GTOS has been shown to underestimate mortality, the STOP criteria may not accurately predict early mortality and futility. This is due to patients with TBI and severe hemorrhage being considered together in the PROPPR (Pragmatic, Randomized Optimal Platelet and Plasma Ratios) study, which provided the foundation for the STOP data [4,8,9]. Therefore, much as the GTOS underestimated the effect of severe TBI in the elderly patient with severe concomitant extracranial hemorrhage, the STOP criteria, which do not consider the presence of TBI as a confounding variable in the estimation of the likelihood of mortality, may overestimate the likelihood of survival in elderly patients in hemorrhagic shock who suffer concomitant TBI [8,9].

The literature has already anticipated these findings with the observation that younger patients with penetrating injury in hemorrhagic shock can survive even after massive volumes of blood have been consumed [4,10,11]. Therefore, it would be useful to propose a triage system where patients who have a greater likelihood of successful resuscitation—that is, the younger patient with severe hemorrhagic shock—be provided the opportunity to consume more blood products, in contrast to older trauma patients with extracranial hemorrhage and severe TBI who have a far lower chance of a good outcome [12,13]. The past literature highlights the different mortality of those patients older than 65 who have clinical evidence of severe TBI compared to those who are younger and do not have TBI. It should be noted that a recent study shows that the elderly have several comortalities and indicators of poor prognosis in trauma beyond TBI, such as oncological disease and a history of anti-platelet medication [14].

Refining the STOP and GTOS criteria to include TBI could represent a simple bedside tool that could enable the clinician to refine their “quantitative gestalt [12]” (a clinician’s heuristic decision-making process, based on intuition and experience developed over many patient cases, augmented by quantitative data from trials) regarding the termination of resuscitation in patients with severe TBI and extracranial injury, especially if they are over 65, have bilaterally fixed and dilated pupils, and have consumed a large amount of blood products in the first hour [15,16].

Therefore, we applaud Jeon et al. for their observation and reaffirmation that GTOS underestimates mortality by the imperfection of not considering TBI as a discrete comorbid factor. Based on the authors’ conclusions, future studies which search for reliable early predictors of futility should begin with the presence or absence of severe TBI, bilaterally fixed and dilated pupils, age, and the number of blood products consumed in the first hour. An example of an algorithm that approaches this logic is the Futility of Resuscitation Measure (FoRM). The FoRM has shown that polytrauma patients with many comorbidities will have a high probability of mortality. These comorbidities are elderly age (70–80 years old), hypotension (a single episode of systolic blood pressure < 50), the early use of vasopressors, aggressive blood transfusion (6–10 units pRBC within the first 4 h), TBI (GCS 3-8), and abnormal head CT (showing midline shift). Statistically, such patients will have a mortality percentage of >95% and an AUROC = 0.86 (*p* < 0.001) [12]. We hope, therefore, that a modification of the GTOS, such as the authors themselves have proposed, would be precisely the type of tool that could be added to the clinical and laboratory parameters such as the STOP parameters in order to predict futility and to spare the dwindling resources of blood components through hemovigilance and blood component stewardship.

As pointed out above, there has been controversy regarding the ability of the number of units transfused per hour to predict futility in severely bleeding trauma patients. Specifically, in patients without TBI, particularly young patients without TBI, transfusion cutoff points seem not to reliably predict certain death. However, a severely bleeding multiple-trauma patient, older than 65 years of age, with severe TBI and continued failure to respond to resuscitation may in fact be a candidate for using transfusion cutoff points for determining futility, as suggested by Bhogadi et al. [12,13,15,16,17,18,19,20].

Jeon et al. have provided traumatologists with evidence that a modified version of the GTOS which utilizes parameters collected during the first hours of resuscitation in elderly patients with severe extracranial and traumatic brain injury may serve as an indicator of mortality. Their work represents an important foundation upon which to develop specific criteria which can accurately predict futility in severely bleeding trauma patients. These criteria may enable traumatologists and their blood bankers to consider transfusion cutoff points per hour, as well as other comorbidities that are unique to the elderly population, to accurately define futility and therefore preserve scarce blood products and prevent wastage [21].

## Abbreviations

The following abbreviations are used in this manuscript:

GTOS	Geriatric Trauma Outcome Score
TBI	Traumatic Brain Injury
BIG	Brain Injury Guidelines
ISS	Injury Severity Score
STOP	Suspension of Transfusion and Other Procedures
PPV	Positive Predictive Value
ROSC	Return Of Spontaneous Circulation
GCS	Glascow Coma Scale
TEG	Thromboelastography
PROPPR	Pragmatic, Randomized Optimal Platelet, and Plasma Ratios
FoRM	Futility of Resuscitation Measure
pRBC	Packed Red Blood Cells
AUROC	Area Under the Receiver Operating Characteristic Curve
